# Perspective: Humanized Pig Models of Bladder Cancer

**DOI:** 10.3389/fmolb.2021.681044

**Published:** 2021-05-17

**Authors:** Natália Vieira Segatto, Camila Bonemann Bender, Fabiana Kommling Seixas, Kyle Schachtschneider, Lawrence Schook, Noah Robertson, Aisha Qazi, Maximillian Carlino, Luke Jordan, Courtni Bolt, Tiago Collares

**Affiliations:** ^1^ Postgraduate Program in Biotechnology, Cancer Biotechnology Laboratory, Technology Development Center, Federal University of Pelotas, Pelotas, Brazil; ^2^ Department of Radiology, University of Illinois at Chicago, Chicago, IL, United States; ^3^ Department of Biochemistry and Molecular Genetics, University of Illinois at Chicago, Chicago, IL, United States; ^4^ National Center for Supercomputing Applications, University of Illinois at Urbana-Champaign, Champaign, IL, United States; ^5^ Department of Animal Sciences, University of Illinois at Urbana-Champaign, Urbana, IL, United States; ^6^ Albion College, Albion, MI, United States

**Keywords:** urothelial carcinoma, bladder cancer, large animal model, pig, oncopig

## Abstract

Bladder cancer (BC) is the 10th most common neoplasia worldwide and holds expensive treatment costs due to its high recurrence rates, resistance to therapy and the need for lifelong surveillance. Thus, it is necessary to improve the current therapy options and identify more effective treatments for BC. Biological models capable of recapitulating the characteristics of human BC pathology are essential in evaluating the effectiveness of new therapies. Currently, the most commonly used BC models are experimentally induced murine models and spontaneous canine models, which are either insufficient due to their small size and inability to translate results to clinical basis (murine models) or rarely spontaneously observed BC (canine models). Pigs represent a potentially useful animal for the development of personalized tumors due to their size, anatomy, physiology, metabolism, immunity, and genetics similar to humans and the ability to experimentally induce tumors. Pigs have emerged as suitable biomedical models for several human diseases. In this sense, the present perspective focuses on the genetic basis for BC; presents current BC animal models available along with their limitations; and proposes the pig as an adequate animal to develop humanized large animal models of BC. Genetic alterations commonly found in human BC can be explored to create genetically defined porcine models, including the BC driver mutations observed in the FGFR3, PIK3CA, PTEN, RB1, HRAS, and TP53 genes. The development of such robust models for BC has great value in the study of pathology and the screening of new therapeutic and diagnostic approaches to the disease.

## Introduction

Bladder cancer (BC) represents the most common neoplasia of the urinary tract and the 10th most common type of cancer worldwide (Bray et al., 2018). BC typically arises from the urothelium (around 90% of BC cases), the innermost tissue of the bladder (ASCO, 2020). Thus, urothelial carcinoma (UC) is the most studied type of BC and the focus of the present perspective.

BC can be further divided into non muscle-invasive BC (NMIBC) and muscle-invasive BC (MIBC), according to the degree of invasiveness of cancer cells into the bladder muscle layer. This classification is extremely important since it is directly linked to the aggressiveness of the tumor and, consequently, survival rates and clinical treatments. MIBC tends to be more aggressive and deadlier than NMIBC (ASCO, 2020). However, there are reports of high tumor recurrence rates at NMIBC after initial treatment, a fact that affects up to 70% of patients. These patients presenting with recurrent tumors have a 10–20% risk of progressing to a more aggressive tumor type leading to muscle invasion (Kaufman et al., 2009). The need for lifelong surveillance, high rates of disease recurrence resulting in long periods of treatment, and resistance to chemotherapy make BC one of the malignancies with the highest cost of lifelong treatment per patient (Sievert et al., 2009). Besides, resistance to the current chemotherapies and immunotherapies highlights the need for more effective treatments for BC. In this regard, biological models capable of recapitulating the characteristics of BC human pathology and progression are essential in evaluating the effectiveness of potential new therapies.

## The Genetic Basis of Human Bladder Cancer

**FIGURE 1:**
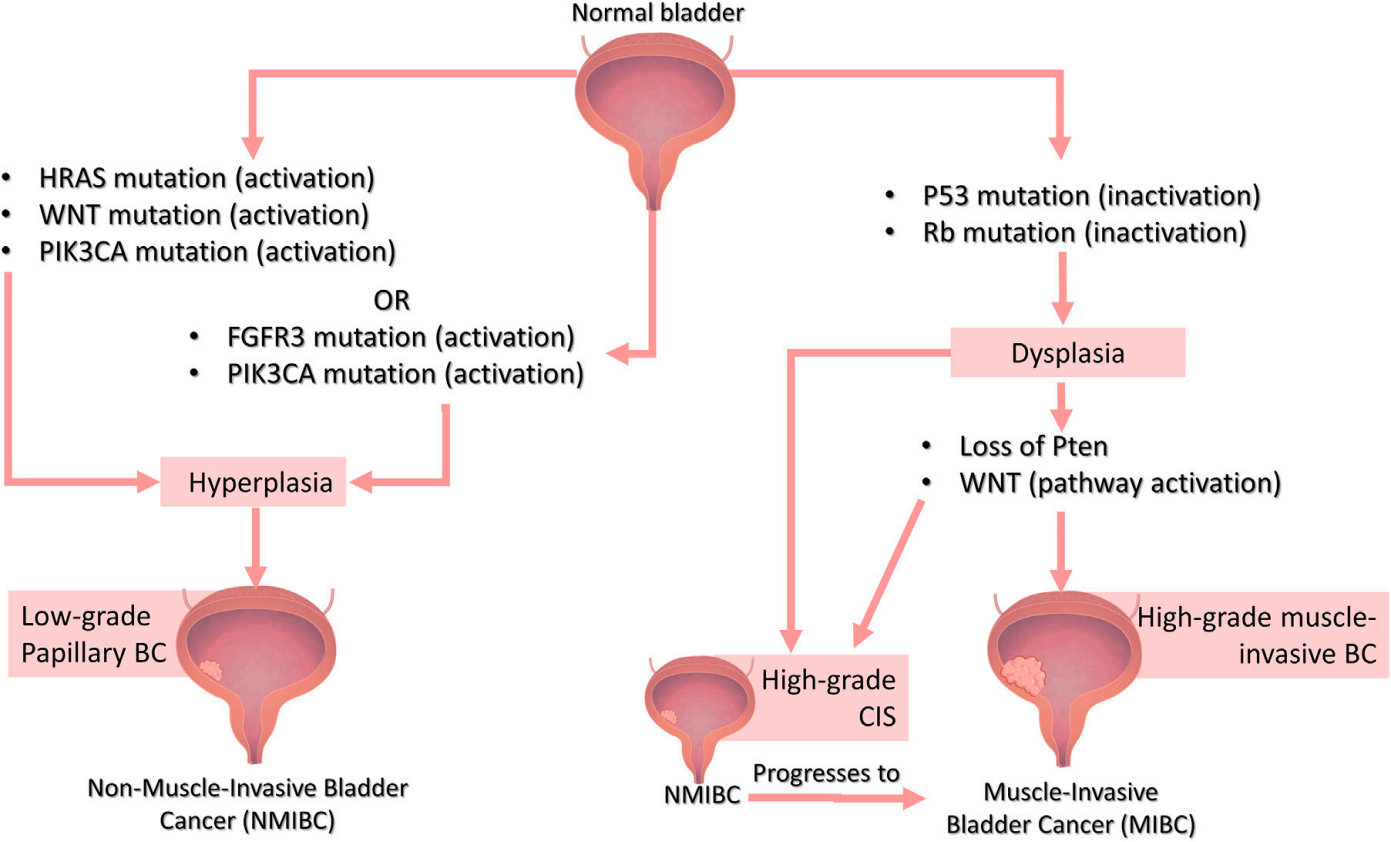
The genetic basis of Bladder Cancer. Distinct genetic pathways are involved in the development of muscle-invasive or non-muscle invasive BC. NMIBC can be sub-classified into low-grade, which are mainly superficial (papillary) tumors that grow into the bladder innermost layers but do not invade the muscle layer, and high-grade, which include a subset of superficial tumors as well as carcinoma *in situ* (CIS), which are characterized by a flattened layer of dysplastic cells that is presumed to be the major precursor of muscle-invasive bladder tumors. NMIBC low-grade papillary lesions are frequently associated with either activating mutations in RAS (usually the H-Ras isoform) or FGFR3 gene (these mutations are mutually exclusive, meaning that they do not occur together). On the contrary, FGFR3 and PIK3CA mutations usually co-occur in NMIBC. The Wnt pathway can also be involved in NMIBC when in combination with RAS pathway activation. In MIBC lesions, as the name suggests, the cells invade the adjacent muscle layer of the bladder and, in some cases, progresses to metastatic tumors. The most commonly found mutations in high-grade lesions such as non-muscle-invasive CIS or MIBC are loss of p53 and RB function. Loss of PTEN function and activation of the Wnt signaling pathway are also related to high-grade tumors especially MIBC. Bladder image obtained from Biorender (https://biorender.com/)

Genetic attributes highly influence the characteristics of UC. Thus, it is important to understand the genetic bases of human BC to create models that closely recapitulate human BC genotypic signatures and develop models with phenotypic and pathogenic features characteristic of human BC to truly recapitulate human BC tumorigeneses. The most prevalent genetic alterations occur in fibroblast growth factor receptor 3 (FGFR3), Phosphoinositide-3-kinase catalytic alpha subunit (PIK3CA), retinoblastoma 1 (RB1), tumor protein 53 (TP53) and HRas Proto-Oncogene (HRAS) genes, with TP53 and FGFR3 being the most common (Knowles, 2006; Humphrey et al., 2016). Molecular changes differ markedly between invasive and non-invasive lesions. Current evidence suggests there are two distinct genetic pathways for the NMIBC and MIBC subtypes (Figure 1), which can be explored to create genetically defined BC models with different degrees of invasiveness, depending on the target-genetic modifications.

Rb and TP53 mutations are rare in superficial (papillary) tumors, but they often occur in invasive lesions. Mutations or deletions in the TP53 gene, for example, are found in around 70% of muscle-invasive UC (Sidransky et al., 1991; Fujimoto et al., 1992; Knowles, 2006) and mutations in the Rb gene is found in 34–37% of invasive tumors (Cordon-cardo et al., 1992; Knowles, 2006). Loss of PTEN function has also been proposed to play a role in MIBC (McConkey et al., 2010). Deletions and mutations of the RAS gene (specially HRAS) and FGFR3, however, occur in most superficial non-invasive papillary tumors, but in a small subset of invasive BC (Knowles, 2006). However, studies indicate that FGFR3 and RAS mutations are mutually exclusive (do not occur together) (Jebar et al., 2005; Kompier et al., 2010; Juanpere et al., 2012), on the contrary of FGFR3 and PIK3CA mutations that usually co-occur in NMIBC (Kompier et al., 2010; Juanpere et al., 2012). Besides, activation of the Wnt signaling pathway along with PTEN loss is related to invasive tumors, while Wnt and RAS pathways activation lead to the development of non-invasive tumors that depend on MAPK signaling (Ahmad et al., 2012; Figure 1).

## Current Animal Models of BC

Animal models of BC support our general understanding of disease and the evaluation of new therapies to translate the preclinical knowledge into effective improvements in patient care. Therefore, efforts are constantly made to modify and optimize BC models in order to ideally represent the disease in question. Pre-requisites of successful BC models include: a) tumors of urothelial origin; b) mimic the pathogenesis of human BC; c) orthotopic tumors; d) present phenotypic and genotypic alterations similar to those found in human BC, while preserving inter-patient heterogeneity; e) immunocompetent animal host (to test immunotherapies); f) preferably, it must be economically viable and easy to handle; g) tumors develop rapidly; and h) provide reproducible and translatable results (Ding et al., 2014). In the following section, we will discuss currently used animal models of BC.

### Transplantable Mouse Models

Murine models are the most widely used BC models, mainly due to their small size, known genetics, ease of handling, and low cost (Kobayashi et al., 2015). Transplantable graft models are obtained through the inoculation of tumor cells cultured *in vitro* in rodents. Xenograft models are established through the transplantation of human urothelial cancer cells in immunodeficient mice (Russell et al., 1986), while syngeneic grafts consist of the inoculation of murine cells in immunocompetent rodents (Chade et al., 2008). A limitation of xenograft models is the impossibility to test immunotherapies since the host animal does not have an effective immune system. Contrary to xenograft BC models, syngeneic grafts use immunocompetent mice which allow testing immunotherapies. However, one disadvantage is the use of murine cells instead of humans. Orthotropic tumors are generated by the intravesical inoculation of cells (human or murine) directly into the bladder and mimic the behavior of human BC more closely than heterotopic models, in which cells are usually inoculated subcutaneously on the flank or rear of the animal. Therefore, it is expected that experimental results generated from the orthotopic model have greater relevance than from heterotopic models (Bibby, 2004).

Literature reports indicate that transplantable graft models in general present problems related to cells cultured *in vitro* for a long time, which tend to differ from the original tumor in terms of morphology, phenotype, or growth patterns since high passage rates are associated with increased spontaneous mutations, senescence and unnatural *in vitro* selection processes (Reznikoff et al., 1994; Gildea et al., 2000; Cristofalo et al., 2004). In addition, they present the disadvantage of not mimicking early BC development, since cancer cells are simply injected into the animal instead of going through the process of tumor formation and progression.

### Carcinogen Induced BC Mouse Models

N- [4- (5-nitro-2-furyl) -2-thiazolyl] formamide (FANFT) (Fujita et al., 1988), N-butyl-N- (4-hydroxybutyl) -nitrosamine (BBN) (Akagi et al., 1973) and N-methyl-N-nitrosourea (MNU) (Hicks and Wakefield, 1972) are chemicals most widely used to experimentally induce urothelial tumors. Carcinogen induced models mimic the occupational exposures that usually lead to the development of BC and provide readily available reproducible models of the BC pathogenesis. However, they have a long tumor induction period and human contact with the carcinogenic substance is harmful.

### Genetically Engineered Mouse Models

GEMM carry mutations in oncogenes and/or tumor suppressor genes related to BC tumorigenesis. Several GEMM of UC use the uroplakin II (UPII) promoter, which consists of a group of proteins related to the differentiation of urothelium and promote urothelium-specific expression (Tian et al., 2015). The first GEMM for BC had an SV40-mutated gene using a UPII promoter. SV40 inactivates both p53 and Rb pathways, and the animals developed highly invasive UC (Grippo and Sandgren, 2000). However, transgenic mice null for both P53 and Rb were extremely sensitive to BBN induced carcinogenesis but did not develop spontaneous tumorigenesis, suggesting that these mutations were insufficient to initiate invasive UCC by itself but are related to progression and invasiveness (He et al., 2009). Invasive BC was generated in transgenic mice bearing p53 and Pten conditional knockouts mediated by the UPIIIa promoter (Saito et al., 2018). To model NMIBC, mice were engineered to activate the PI3K pathway by β-catenin and Pten alterations (Ahmad et al., 2011a) or the MAPK pathway by β-catenin and HRAS alterations (Ahmad et al., 2011b) in the urothelium using a UPII Cre construction.

However, generation of GEMM is a laboratory technique, and only a small proportion of the animals produced exhibit the desired genotypic characteristics. Besides, mouse models as a whole display dissimilarities toward humans in drug metabolism (Swanson et al., 2004), size, cancer genetics (Rangarajan and Weinberg, 2003), and metabolic rates (Rangarajan and Weinberg, 2003), with studies showing large differences in drug metabolism and xenobiotic receptors, such as CYP enzymes (Martignoni et al., 2006).

### Canine Spontaneous Muscle-Invasive UC Model

Dogs develop spontaneous-muscle-invasive UC in a similar way to humans. The similarities include physiological age of onset of the lesion, clinical symptoms, cellular and pathological characteristics (including high degree, tumor heterogeneity, and local invasion), biological behavior (such as sites and frequency of metastases), response to chemotherapy (e.g. cisplatin treatment) and shared molecular targets. The canine’s large size, compared to rodents, makes many medical procedures technically feasible (Sommer et al., 2018). Besides, companion dogs in general are attractive translatable models because they are genetically similar to humans and share environmental exposures with their human owners (Alvarez, 2014; Fenger etl al., 2016). However, spontaneous UCs are considered rare in dogs, representing only 2% of all canine cancers (Norris et al., 1992), even though there are breeds associated with high risk for BC, such as Scottish Terriers (Knapp et al., 2020). Since they are not inducible, scientists have to rely on the spontaneous development of BC in dogs to perform co-clinical canine trials (Knapp et al., 2020). Even though spontaneous-muscle-invasive UC canine models are good BC models, they do not fulfill the needs of the scientific community which craves readily available inducible models.

In this context, we believe the future directions in the research and development of BC models will prioritize the generation of genetically defined inducible models bearing mutations commonly found in human BC to create humanized models of the disease. Large animals arise as attractive platforms to develop such models which would be highly valuable in preclinical trials. In this sense, we propose the swine as an adequate animal to develop humanized large models of BC and highlight the potential of swine platforms in modeling BC.

## Swine as Biological Models

Pigs are a large-sized animal with the advantage of not raising ethical burdens commonly faced when using non-human primates since pigs are treated as agricultural livestock animals. The benefits of using large animal models include body/organ size and lifespan similar to humans, which allow researchers to use the same tools and techniques applied in the clinic. The potential of swine platforms should thus be explored to model BC. However, these animals have to be genetically engineered, since there are no reports–to our knowledge - of spontaneous BC in swine. Genetically induced tumors have the advantage of representing a wide set of genes, however, it is not certain that these modifications will translate to the physiopathology of the disease, like in spontaneous models.

The porcine urological system is similar anatomically and physiologically to humans. A porcine model of cystitis (inflammation of the bladder) has been developed due to the pig’s urothelial similarities to humans’ (Nielsen et al., 2019), and regular cystitis is positively associated with BC risk (Vermeulen et al., 2015).

The immune system plays a major role in cancer, especially in BC where the gold standard treatment for NMIBC is BCG immunotherapy (Babjuk et al., 2017). The porcine immune system is similar to humans, and they are composed of the same immune cell populations (Meurens et al., 2012). In this sense, pigs are proposed as excellent immune-oncology platforms (Overgaard et al., 2018). Besides, both humans and swine require similar mutations to transform normal cells into tumor cells (Adam et al., 2007). This highlights the potential of genetically modifying oncogenes and tumor suppressor genes related to BC in pigs.

The pig is a valuable preclinical model to predict human response to therapies in clinical trials. They are more predictive of the therapy-response in humans than murine models (Meurens et al., 2012).

As such, pigs have been emerging as suitable biomedical models for several human diseases (Prather, 2013; Schook et al., 2015a). Currently, porcine cancer models have been developed for breast (Luo et al., 2011), colorectal (Flisikowska et al., 2012), intestinal cancer (Callesen et al., 2017), osteosarcoma (Saalfrank et al., 2016) and TP53^R167H^ mutation models (Leuchs et al., 2012). Besides, soft-tissue sarcoma (Schook et al., 2015b; Schachtschneider et al., 2017a), pancreatic ductal adenocarcinoma (Principe et al., 2018) and hepatocellular carcinoma (Schachtschneider et al., 2017b; Gaba et al., 2018) have been developed in swine through injection of an adenoviral vector encoding Cre recombinase in distinct sites in the Oncopig Cancer Model (OCM), a genetically defined model containing two driver mutations (TP53^R167H^ and KRAS^G12D^) and a CreLoxP system developed by our group.

We are currently investigating the *in vitro* treatment-response of swine and human UC cells to chemotherapeutics and BCG immunotherapy. So far, porcine cells mimic the response of human cells, providing similarities between cytotoxicity levels and death pathways following treatment exposures (data not published). However, it is necessary to acknowledge the porcine models’ limitations. Due to its bigger size, it is more expensive to shelter swine than mice models (Segatto et al., 2017), and it is more laborious to develop genetic modifications.

### Perspective: Humanized Swine Models of BC

Humanized models consist of engineered models to express human gene products. Pigs represent an ideal animal to develop personalized tumors due to their size, anatomy, physiology, metabolism, immunity, and genetics similar to humans (Groenen et al., 2012; Flisikowska et al., 2016; Schachtschneider et al., 2017c; Segatto et al., 2017; Xu et al., 2019). Approaches to create this highly valuable humanized BC model would be the addition of a mutated pRB1 gene and uroplakin II promoter to the OCM construction. Defects in the pRb tumor suppressor pathway are commonly found in muscle-invasive UCs along with mutated TP53 (Cordon-Cardo, 2004). The conditional inactivation of RB1 gene alone fails to accelerate urothelial proliferation in the mouse because the p53 pathway is triggered as an apoptosis defense mechanism. However, adding a loss of p53 (which is already present in the OCM) in pRB-deficient urothelial cells results in superficial papillary bladder tumors (He et al., 2009).

**FIGURE 2:**
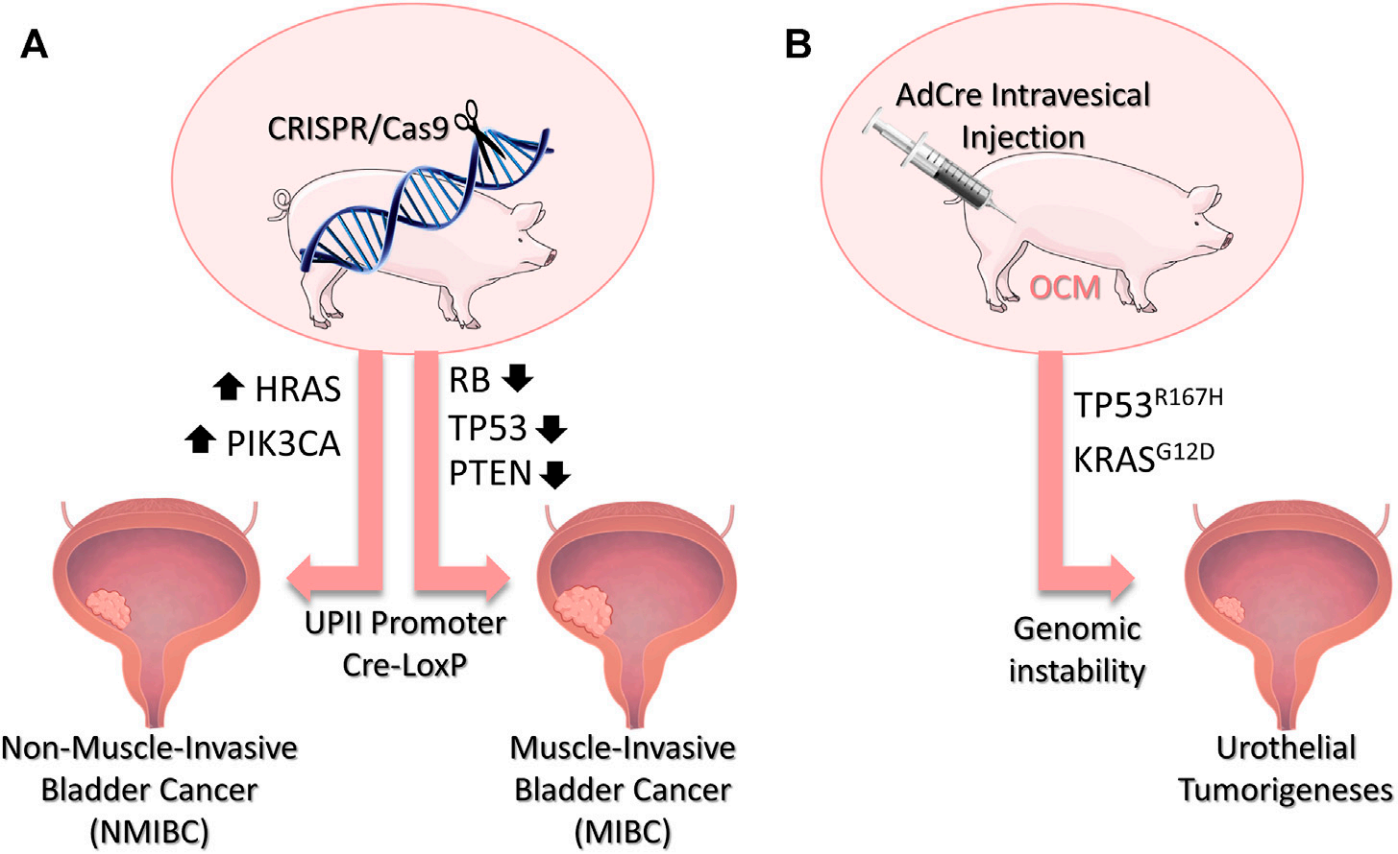
Porcine Bladder Cancer Models. **(A)** Potential genetic modifications in swine to develop bladder cancer models through CRISPR/Cas9 technology. Mutations resulting in the activation of the PIK3CA and HRAS genes could be explored to develop MNIBC, while inactivation of the genes Rb, TP53 and/or PTEN are potentials targets to model MIBC. The addition of UPII promoter and the Cre-LoxP system would allow for a urothelial-induced expression of the target-genes. **(B)** The Oncopig *Cancer* Model (OCM) holds in its genome mutated TP53^R167H^ and KRAS^G12D^ which are activated upon AdCre injection. Intravesical injection of AdCre in the OCM would result in the expression of the transgenes and genomic instability, possibly leading to urothelial tumorigeneses. Bladder image obtained from Biorender (https://biorender.com/)

Other genetic alterations commonly found in human BC to be explored include modifications in the PIK3CA, PTEN (phosphatase and Tensin Homolog), RB1, HRAS, and TP53 genes (Figure 2). Lessons learned from successful GEMM should be used to translate potential tumorigenic genetic alterations to pig models. With the current genome editing technologies available (Schook et al., 2016), such as CRISPR/Cas9, several genes can be engineered in large animals in a much simpler manner to create robust models that recapitulate several genetic features of BC. MIBC could be modeled by inactivation of Rb, TP53 and/or PTEN using the inducible Cre-LoxP in the swine. For NMIBC models, mutations that activate PIK3CA and HRAS could be explored (Figure 2).

Humanized models must also be capable of providing a suitable microenvironment, immune cell response, and tumor invasiveness. There is no data about how these bladder cancer driver mutations would translate in porcine models because no such model has been developed so far. Studies in this matter would be greatly appreciated. However, data from different porcine tumor types may help provide possible answers. A humanized porcine model of intestinal cancer using multiple driver transgenes-KRASG12D, MYC proto-Oncogenec (MYC), simian virus 40 large tumor antigen (SV40LT), and Rb-effectively generated animals with lymph node metastasis (Callesen et al., 2017). Studies of tumor microenvironments demonstrate similarities between the humanized OCM and human cancer genomic landscapes (Schachtschneider et al., 2017c). Besides, OCM tumors have demonstrated to invoke an antitumor immune response dominated by cytotoxic T cells alongside immunoregulatory molecules, creating a tumor microenvironment containing tumor cells along with immune cells (Overgaard et al., 2018b). Recently published data has also shown the oncopig tumor holds a heterogeneous population of tumor hepatocellular carcinoma cells. The results indicate the presence of intratumor heterogeneity resulted from the accumulation of somatic mutations in distinct tumor cells as commonly observed in human HCC (Gaba et al., 2020). Finally, the OCM showed that expression of TP53^R167H^ and KRAS^G12D^ generated tumors that recapitulated transcriptional characteristics found in human soft tissue sarcomas (Schachtschneider et al., 2017a). As such, we also hypothesize that the intravesical inoculation of AdCre in the OCM could generate porcine bladder tumors with histopathological characteristics similar to human BC (Figure 2).

## Discussion

Research and development success of new BC treatments highly depends upon using realistic models. Herein, we propose the swine as an adequate animal to develop humanized large models of BC. The porcine large size is ideal for performing surgical procedures and utilizing urological instruments similar to those used clinically, which is particularly helpful for BC studies where surgery is performed in more than 90% of the patients (ASCO, 2020). Their immune system closely relates to humans which makes it a great option for testing immunotherapies such as BCG (*Bacillus* Calmette–Guérin), the gold standard treatment for NMIBC (Babjuk et al., 2017).

To our knowledge, no BC humanized models have been developed in swine platforms. It is an area of huge opportunities. The development of such robust models holds great value in the study of pathology and the screening of new therapeutic or diagnostic approaches for the disease. BC models available today fail to provide to the scientific community a model that is both genetically inducible and large-sized, which is, in our opinion, essential to fulfilling the need of pharmaceutical trials and physicians aiming to approve new drugs or develop new surgical skills. However, our goal is not to promote the academic standing of pig models, but to place them in their ideal position within oncology research. Mouse models represent a useful tool to study the genetic aspects of the disease, while spontaneous canine models provide a translatable model for medical research. Humanized pigs, however, may be a suitable model for the scientific community based on its tractability. We must recognize that no model is perfect, however, different models can be useful for different purposes, and they all complement each other.

## Data Availability

The original contributions presented in the study are included in the article further inquiries can be directed to the corresponding author.
